# PERCEPTIONS OF OLDER ADULTS ON YOGA-BASED INTERVENTIONS FOR MULTIMORBIDITY IN MYSORE, INDIA

**DOI:** 10.1093/geroni/igad104.3518

**Published:** 2023-12-21

**Authors:** Kiranmayee Muralidhar, Purnima Madhivanan, Anil Behal, Vijaya Srinivas, Nagalambika Ningaiah, Poornima Jaykrishna, Rahul Shidhaye, Karl Krupp

**Affiliations:** Public Health Research Institute of India, Mysore, Karnataka, India; University of Arizona, Tucson, Arizona, United States; Philadelphia, Pennsylvania, United States; Public Health Research Institute of India, Mysore, Karnataka, India; Public Health Research Institute of India, Mysore, Karnataka, India; Public Health Research Institute of India, Mysore, Karnataka, India; Pravara Institute of Medical Sciences, Loni, Maharashtra, India; Mezcoph, Tucson, Arizona, United States

## Abstract

Globally, over half of older adults suffer from ≥2 chronic diseases. In India, proven exercise interventions are limited by individual, social, environmental and policy-level factors. We aimed to qualitatively explore perceptions about yoga interventions for healthy aging in Mysore, India. In collaboration with the Community Advisory Board, we developed a qualitative assessment of knowledge, attitudes and behaviors around yoga among seniors. Between August-September 2022, we conducted focus group discussions (FGDs) with older adults, after obtaining informed consent. Interviews were recorded, transcribed, and translated from the local language Kannada. Data were coded manually, and emerging themes were identified. We conducted 4 FGDs of 7-8 participants each; 14 male and 13 female older adults participated, and their average age was 66.1±4.3 years; 88.9% had at least 10 years of education. Most participants expressed interest in yoga for health maintenance, specially mentioning its Indian roots, but did not prioritize it for routine practice. Yoga practice was also a means to reduce social isolation for many. Reported challenges in adopting yoga practice included inaccessibility to instructors and dependence on family members. Cultural barriers especially affected women, including ageism and gender role expectations. Women reported hesitancy to express their desire to learn yoga due to perceived negative reactions from their younger family members and their community. Promoting health promotion practices among older Indian adults is challenging primarily due to cultural norms and changing family dynamics with age. Promoting healthy behaviors among them through yoga will first involve family education and consent.

